# Diplopia and Optic Disc Edema as the Ocular Manifestations of COVID-19 in a Seven-Year-Old Child

**DOI:** 10.1155/2022/8431692

**Published:** 2022-06-17

**Authors:** Anna Rzeszotarska, Marta Pawlak, Chmielarz-Czarnocińska Anna, Justyna Szczapa-Jagustyn, Jarosław Kocięcki, Joanna Siwiec-Siwiec-Prościńska, Anna Gotz-Więckowska

**Affiliations:** Department of Ophthalmology, Poznan University of Medical Sciences, Poznan, Poland

## Abstract

Serious ocular complications due to SARS-CoV-2 disease in children are rare. Herein, we present a case report of a seven-year-old patient, who was diagnosed with pediatric inflammatory multisystem syndrome (PIMS) due to COVID-19 and developed the ocular manifestations comprising diplopia and binocular optic disc edema. The patient condition improved within few weeks without any ocular sequels so far.

## 1. Introduction

The prevalence of severe forms of coronavirus disease 2019 (COVID-19) in children is very low, accounting for only 1-2% of detected cases worldwide [1–3]; however, some pediatric patients may develop a life-threatening pediatric inflammatory multisystem syndrome (PIMS). There are only a limited number of publications regarding ocular findings caused by COVID-19 in pediatric patients [4–6].

This is a case report of a child diagnosed with PIMS concomitant with diplopia and bilateral optic disc edema as the ocular manifestations of COVID-19.

## 2. Case Report

In February 2021, a seven-year-old female patient was hospitalized in the Pediatric Department with a suspicion of appendicitis. During hospitalization, the patient developed cardiological complications (abnormalities in myocardial contractility, right ventricle enlargement) and dermal and mucosal changes, including conjunctivitis. A laboratory test for bacterial and viral infections (*Yersinia, Salmonella, Shigella, Escherichia coli, Campylobacter,* SARS-CoV-2) was performed. All the tests turned out to be negative except for anti-SARS-COV-2 IgG, which was positive. Eventually, considering the overall clinical picture, the patient was diagnosed with PIMS. Ten days after admission, she started complaining of diplopia.

**Figure 1:**
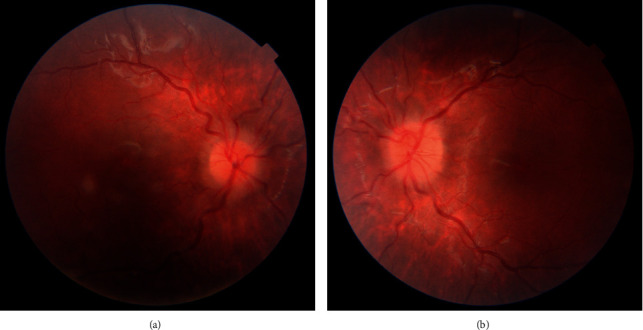
The fundus of the right (a) and of the left (b) eye at the baseline visit—optic disc edema and vessels tortuosity.

During the first ophthalmological consultation, the best corrected visual acuity was 0.0 logMAR in both eyes. The intraocular pressure measured with I-Care was within normal limits, i.e., 11.6 and 11.3 mmHg in the left and right eye, respectively. The primary eye position and motility were also normal, whereas the prism cover test (PCT) showed an esophoria of 16 prismatic diopters. The Worth test performed at a distance revealed diplopia. Stereo testing using the Distance Randot® Stereotest was abnormal, whereas the near Randot test result was 20 seconds of arc. Pupillary reaction, both direct and indirect, and color vision, tested with Panel D-15, were normal. The anterior segment assessment did not show any abnormalities; nevertheless, the ocular fundus exam revealed optic nerve head edema and vessel tortuosity in both eyes (Figures 1(a) and 1(b)). The brain MRI did not show any changes in the orbits, brain, or optic nerves, optic chiasm, or optic tracts. However, the visual evoked potential revealed bilateral dysfunction in the visual pathway, a broad P100 wave, and prolongation of P100 mean latency; the P100 amplitude was within normal limits. The patient was treated with 150 mg of acetylsalicylic acid per day, as recommended by cardiologists.

**Figure 2:**
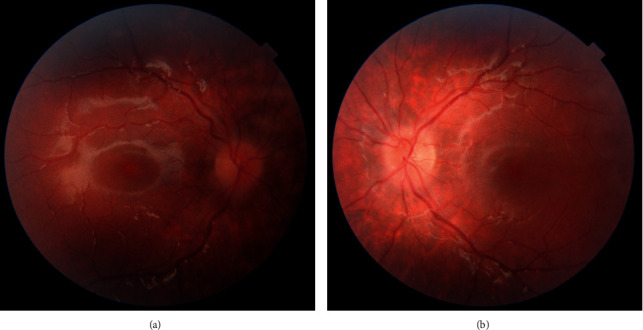
The fundus of the right (a) and of the left (b) eye after three weeks—resolution of the optic disc edema.

Three weeks later, the patient reported resolution of diplopia. Her visual acuity did not deteriorate. The fundus examination and OCT RNFL revealed remission of the optic nerve head edema (Figures 2(a) and 2(b)), and the Worth Four Light test confirmed normal binocular response. In addition, the PCT revealed an improvement of eye alignment to an esophoria of 4 prismatic diopters. Three months later, the electrophysiology parameters improved. The patient remains in the follow-up of the Pediatric Outpatient Ophthalmologic Clinic.

## 3. Discussion

Conjunctivitis appears to be the most common ocular manifestation of COVID-19 in adults and children [4]. In animals, coronaviruses can cause severe ocular complications including anterior uveitis, retinitis, vasculitis, and optic neuritis [7]. Several reports have also described such changes in humans. François et al. published a case report of a 50-year-old woman with SARS-CoV-2 with severe neuropathy and panuveitis [8]. Alcalde et al. presented a case series of ocular findings in children diagnosed with COVID-19 [5]. The first was the case of an 11-year-old patient with retinitis, and the second case related to a 13-year-old boy diagnosed with postinfectious right optic neuritis. Baccarella et al. [6] presented a case series similar to ours of two patients with increased intracranial pressure and concomitant diplopia and abducens palsy in course of SARS-CoV-2 infection.

We are aware of limitations of the presented case. The main limitation is the fact that we did not perform the lumbar puncture for opening pressure or cerebrospinal fluid (CSF) analysis. Also, we present only a single case. Admittedly, our patient had similar symptoms to those presented by Baccarella et al. in their case series; however, we also contributed in electrophysiological presentation.

In the presented case, all of the ocular symptoms subsided and, most importantly, the patient recovered with no serious ophthalmological consequences. Despite the fact that severe ocular complications are rare, practitioners should consider COVID-19 in the differential diagnosis of optic disc edema or sudden onset of diplopia in both children and adults.
